# *Angiostrongylus vasorum*: Experimental Infection and Larval Development in *Omalonyx matheroni*

**DOI:** 10.1155/2011/178748

**Published:** 2011-05-31

**Authors:** L. R. Mozzer, L. C. Montresor, T. H. D. A. Vidigal, W. S. Lima

**Affiliations:** ^1^Departamento de Parasitologia, Universidade Federal de Minas Gerais, Avenida Presidente Antônio Carlos 6627, Caixa Postal 486, 31270-901, Belo Horizonte, MG, Brazil; ^2^Departamento de Zoologia, Universidade Federal de Minas Gerais, Avenida Presidente Antônio Carlos 6627, Caixa Postal 486, 31270-901, Belo Horizonte, MG, Brazil

## Abstract

The susceptibility and suitability of *Omalonyx matheroni* as an intermediate host of *Angiostrongylus vasorum* and the characteristics of larval recovery and development were investigated. Mollusks were infected, and from the 3rd to the 25th day after infection, larvae were recovered from groups of 50 individuals. The first observation of L2 was on the 5th day, and the first observation of L3 was on the 10th day. From the 22nd day on, all larvae were at the L3 stadium. Larval recovery varied from 78.2% to 95.2%. We found larval development to be faster in *O. matheroni* than in *Biomphalaria glabrata*. Our findings indicate that this mollusk is highly susceptible to *A. vasorum*. Infective L3 were orally inoculated into a dog, and the prepatent period was 39 days. This is the first study to focus on *O. matheroni* as an intermediate host of *A. vasorum*.

## 1. Introduction

The nematode *Angiostrongylus vasorum *is a parasite of wild and domestic canids. Adult worms are found in the right ventricle, pulmonary artery, and its branches, where sexual reproduction and oviposition take place. The first-stage larvae (L1) hatch in the alveoli, migrate up the bronchial tree, and are swallowed and then excreted into the environment along with the host feces. Infection frequently leads to pneumonia, loss of racing performance, coughing, and anemia [1]. Severely infected dogs may develop cardiac insufficiency and pulmonary fibrosis, followed by weight loss, hemorrhagic diatheses, and death [2, 3]. Several terrestrial and aquatic mollusks may act as intermediate hosts [4–7]. The genus *Omalonyx *(Pulmonata: Stylommatophora) belongs to the family Succineidae, which is composed of hermaphroditic terrestrial pulmonates that are morphologically diverse. *Omalonyx *sp. have a reduced flat shell and slug-like body, and they can be found in humid soil and in macrophytes [8–10]. They have a broad geographical distribution east of the Andes in South America and in the Lesser Antilles Islands [9], including localities where *A. vasorum *is known to occur [11, 12]. These mollusks are important intermediate hosts of the trematode *Leucochloridium *[13–15] and are able to support the life cycle of *Angiostrongylus costaricensis *in the laboratory [12]. There is no record of *Angiostrongylus *naturally infecting *Omalonyx*. This investigation aimed to evaluate the susceptibility and suitability of *Omalonyx matheroni *as an intermediate host of *A. vasorum *and to analyze the parasite's larval development from L1 to L3. Studies on the development of *A. vasorum *in different hosts contribute to the understanding of the parasite's biology and of the host-parasite relationship.

## 2. Methods

### 2.1. Mollusks

young individuals (from 25 to 30 days old) of *O. matheroni *(*n* = 1150) measuring from 9 to 14 mm in length, raised under laboratory conditions, and from parental specimens from Pampulha Lake in Belo Horizonte, Minas Gerais State, Brazil were employed in this trial.

### 2.2. Parasites

*A. vasorum *L1 were obtained from the cycle maintained in the laboratory using successive passages in snails (*Biomphalaria glabrata*) and dogs (*Canis familiaris*). This strain was isolated from a dog in Caratinga, Minas Gerais State [11].

### 2.3. Mollusk Infection

The feces of infected dogs was collected, and L1 were recovered by the modified Baermann apparatus [16]. After 24 hours of fasting, mollusks were individually placed in polystyrene culture test plates with 24 wells of 15 mm diameter (TPP—Techno Plastic Products, Switzerland) and fed with 250 L1 on a fragment of lettuce (approximately 15 mm diameter). After 8 hours, they were transferred to a plastic container (20 × 12 cm) with 250 mL of dechlorinated tap water and wood pieces. Groups of 10 individuals were kept in these containers during this trial. They were maintained at room temperature (25 to 27°C) and were fed on lettuce. Larvae that stayed in each test plate were counted and subtracted from the total amount offered to each individual. It is assumed that this is the number of larvae that entered each individual and calculate, for groups of 50 individuals: the number of larvae that entered the hosts, the percentage of larvae that entered the hosts, and the percentage of larvae recovered (Table 1).

### 2.4. Larval Development

From the 3rd to the 25th day after infection, larvae (L1, L2 and L3) were recovered from groups of 50 mollusks in a Baermann apparatus and fixed in Railliet-Henry at 60°C for quantification and identification of the larval stage [7]. Larval stage was identified based on published descriptions [7, 11, 17].

### 2.5. Dog Infection

To verify whether L3 from *O. matheroni *(21 days after infection) were infective, 1000 larvae were orally inoculated into a male mongrel dog weighing 10 kg born in the breeding facilities of the Universidade Federal de Minas Gerais, under the management systems on animal well-being and according to the ethics committee of the university (CETEA/UFMG). After the 20th day of infection, feces was collected daily for parasitological investigation of the presence of larvae.

## 3. Results

### 3.1. Mollusk Infection

*O. matheroni *was susceptible to the infection. Larvae (L1, L2 and L3) were recovered from the 3rd to the 25th day after infection. After 8 hours of contact, 95.2 to 97.8% of the larvae had penetrated the mollusks (Table 1).

### 3.2. Larval Development

The amount of larvae recovered each day is presented in Table 1. Mean L3 recovery is reported as the number of L3 recovered divided by the number of L1 that penetrated the host. These proportions were between 78.2% and 95.2%.

### 3.3. Dog Infection

**Figure 1:**
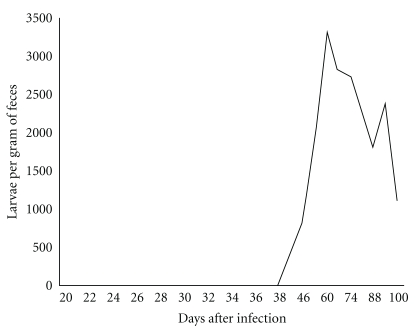
Number of larvae per gram of feces recovered from an experimentally infected (1000 L3) *Canis familiaris*.

Larvae were first detected in the feces in the 39th day (512 per g of feces) and increased until the 60th day (3320 per g of feces). This increase was followed by a gradual decrease that reached 1120 larvae per gram of feces on the 100th day (Figure 1). During this 100-day period, the amount of larval release varied, but larvae were never absent.

## 4. Discussion

Nematodes of the genus *Angiostrongylus*, including the species *A. vasorum, *can infect a wide spectrum of intermediate hosts of the class Gastropoda [18]. This system thus represents an interesting experimental model for the study of the host-parasite relationship. The susceptibility of a mollusk to a protostrongylid has been defined in terms of L1 penetration capability, the possibility of L3 development and time required to complete larval development [19, 20]. The present investigation demonstrates the susceptibility and suitability of *O. matheroni *as an intermediate host of *A. vasorum*. The percentage of L3 recovery in *O. matheroni *varied from 78.2% to 95.2%. The high percentage of larval recovery confirms our findings and indicates that this mollusk is highly susceptible to *A. vasorum*. Infective L3 recovered from these mollusks developed into fertile adults. L1 were observed in the feces of the infected dog. 

Several factors influence the larval development of protostrongylids in the intermediate host such as environmental conditions (i.e., temperature) and biological conditions (i.e., hosts species and age) [21–24]. Geritcher [21] emphasized that among the environmental factors affecting the development of protostrongylid larvae in snails, the most important is temperature [21]. Low temperature (18 to 20°C) increases the time of development of the larvae, whereas high temperatures accelerate their development (25 to 28°C), as observed for the genus *Angiostrongylus *[17, 25, 26]. In this work, we observed that larval development of *A. vasorum *is faster in *O. matheroni *than in other known intermediate hosts [17, 27]. This conclusion is based on comparisons with data that is available in the literature. Experimental infection of several species of terrestrial mollusks (maintained at 18 to 23°C) allowed the first observations of L3 on the 16th and 17th day after infection [27]. Such low temperatures increase the time of larval development, and we are focusing our discussion on works that were performed at higher temperatures (25 to 28°C). In a trial where *B. glabrata *was maintained at 25 to 27°C, L2 were recovered between the 7th and 8th day after infection and L3 on the 14th and 15th [17]. Our results for *O. matheroni *demonstrated that L2 can be observed for the first time on the 5th day after infection and L3 can be observed for the first time on the 10th day. Furthermore, after 21 days, almost all larvae recovered were L3. The exploitation of hosts' immune response by the parasite was discussed by Damian [28], and the encapsulation of *A. costaricensis *in veronicellidae slugs has been considered an example of such a process [29]. 

Larvae were observed in the feces of the experimentally infected dog 39 days after infection. These results corroborate those of Bessa et al. [7], Oliveira-Júnior et al. [30], and Barçante et al. [16], who observed a prepatent period varying from 28 to 108 days afterinfection. 

In view of the high reproductive rates of *O. matheroni *and the feasibility of laboratory rearing (accelerated larval development, efficient larval recovery, and larval viability), we consider such mollusks very useful for the maintenance of the *A. vasorum *cycle in the laboratory. Moreover, this mollusk is also an interesting experimental model for studies on the host-parasite relationship of *A. vasorum *and its intermediate hosts.

## Figures and Tables

**Table 1:** Larval recovery from groups of 50 *Omalonyx matheroni* experimentally infected with 12500 L1 of *Angiostrongylus vasorum* (250 L1 per mollusk).

DPI	Number of larvae that entered the hosts	Percentage of larvae that entered the hosts (%)	Number of larvae recovered	Percentage of larvae recovered (%)	Number of L1 recovered	Number of L2 recovered	Number of L3 recovered
3	11997	95.9	10009	83.4	10009	0	0
4	11900	95.2	9300	78.2	9300	0	0
5	12038	96.3	10113	84.0	9364	749	0
6	11937	95.5	9750	81.7	5675	4075	0
7	12149	97.2	10154	83.6	6417	3737	0
8	12225	97.8	11635	95.2	7446	4189	0
9	11987	95.9	9942	82.9	5349	4593	0
10	12187	97.5	10207	83.8	4001	5675	531
11	12006	96.1	10012	83.4	1782	7028	1202
12	12033	96.3	10024	83.3	1484	6435	2105
13	12076	96.6	10131	83.9	932	6768	2431
14	11993	95.9	9974	83.2	0	6413	3561
15	12207	97.7	11018	90.3	0	5246	5772
16	11972	95.8	9891	82.6	0	3858	6033
17	11984	95.9	9923	82.8	0	2322	7601
18	11905	95.2	9539	80.1	0	1784	7755
19	11954	95.6	9840	82.3	0	1081	8759
20	12075	96.6	10116	83.8	0	364	9752
21	11979	95.8	9902	82.7	0	246	9656
22	11910	95.3	9573	80.4	0	0	9573
23	11918	95.3	9727	81.6	0	0	9727
24	11995	95.9	10008	83.4	0	0	10008
25	11911	95.3	9620	80.8	0	0	9620
